# TRIM8 Promotes Epileptiform Activity by Destabilizing the Glucocorticoid Receptor NR3C1 and Enhancing AMPA Receptor Phosphorylation

**DOI:** 10.3390/biomedicines14071425

**Published:** 2026-06-24

**Authors:** Xiaobing Li, Yan Jia, Bo Fang, Min Xu, Xufang Xie, Xi Lu

**Affiliations:** Department of Neurology, The First Affiliated Hospital, Jiangxi Medical College, Nanchang University, Nanchang 330006, China

**Keywords:** epilepsy, TRIM8, NR3C1, protein stability, AMPA receptor phosphorylation

## Abstract

**Background:** The glucocorticoid receptor NR3C1 exhibits antiepileptic properties, but the mechanisms governing its stability during epileptogenesis remain elusive. This study investigated whether the E3 ubiquitin ligase TRIM8 regulates neuronal hyperexcitability and epileptic activity by modulating NR3C1. **Methods:** We established an in vivo epilepsy model via intrahippocampal kainic acid (KA) injection and an in vitro epileptiform model using Mg^2+^-free artificial cerebrospinal fluid in primary hippocampal neurons. The roles of TRIM8 and NR3C1 were assessed using in vivo and in vitro gain- and loss-of-function approaches, alongside co-immunoprecipitation, Western blotting, immunofluorescence and whole-cell patch-clamp recording. **Results**: TRIM8 is significantly upregulated in hippocampal and temporal lobe neurons in epileptic mice. TRIM8 was markedly upregulated in the hippocampal neurons of epileptic mice, inversely correlating with NR3C1 levels. Mechanistically, TRIM8 interacted with NR3C1, promoting its polyubiquitination and proteasomal degradation. This TRIM8-mediated NR3C1 reduction enhanced the phosphorylation of AMPA receptor (AMPAR) subunits GluR1 (Ser831) and GluR2 (Ser880) without affecting total receptor expression. In vitro, TRIM8 overexpression exacerbated calcium dysregulation, neuronal injury, and AMPAR phosphorylation; crucially, concurrent NR3C1 overexpression rescued these effects. In vivo, knockdown of TRIM8 significantly reduced seizure frequency, prolonged the latency to the first Stage III seizure, shortened average seizure duration, and decreased total seizure burden in KA-induced epileptic mice. Electrophysiologically, TRIM8 overexpression significantly increased the frequency of spontaneous action potentials and amplitudes of spontaneous excitatory postsynaptic currents under Mg^2+^-free conditions. Furthermore, in vivo knockdown of TRIM8 attenuated KA-induced seizure severity, restored NR3C1 protein stability, and suppressed aberrant AMPAR phosphorylation in the hippocampus. Triple immunofluorescence staining showed that KA-induced epilepsy increased TRIM8 but decreased NR3C1 immunoreactivity in NeuN^+^ hippocampal neurons, and TRIM8 knockdown reversed these changes. **Conclusions**: TRIM8 acts as a critical driver of epileptiform activity by targeting NR3C1 for degradation, thereby disinhibiting AMPAR phosphorylation and enhancing network hyperexcitability. The TRIM8-NR3C1-AMPAR axis emerges as a previously unrecognized molecular pathway in epileptogenesis, highlighting its potential as a promising therapeutic target for epilepsy.

## 1. Introduction

Epilepsy is a common chronic neurological disorder characterized by highly synchronous abnormal discharges of neurons in the brain. Its pathogenesis is complex, involving multiple aspects such as ion channels, neurotransmitters, synaptic plasticity, and neural network remodeling. Among these, neuronal hyperexcitability represents one of the core pathological features underlying seizure generation and propagation [1]. The α-amino-3-hydroxy-5-methyl-4-isoxazolepropionic acid receptor (AMPAR) is a key molecule mediating fast excitatory synaptic transmission in the brain. Importantly, phosphorylation of AMPAR subunits at specific sites, especially GluR1 Ser831 and GluR2 Ser880, represents a well-characterized molecular mechanism underlying enhanced synaptic strength and is strongly linked to pathological neuronal hyperexcitability [2,3,4,5,6].

TRIM8 is a member of the TRIM (Tripartite Motif) family of proteins. As an E3 ubiquitin ligase, it recognizes specific substrate proteins and mediates their polyubiquitination, thereby targeting them for proteasomal degradation or functional modification [7]. Notably, TRIM8 is the first gene in the TRIM family found to be associated with epileptic encephalopathy. De novo truncating mutations in TRIM8 have been identified in patients presenting with early-onset epilepsy, developmental delay, and focal segmental glomerulosclerosis [8,9,10]. The vast majority of disease-causing mutations are concentrated in the last exon encoding the C-terminal region, which is crucial for protein dimerization and nuclear localization. It is hypothesized that such truncating mutations may escape nonsense-mediated mRNA decay and produce stable products with dominant-negative characteristics [8]. Although transcriptional upregulation of TRIM8 has been reported in some patients with epilepsy, the functional consequences of altered TRIM8 expression in epileptogenesis remain largely unexplored. Given the substrate-specific regulation by E3 ubiquitin ligases, we hypothesized that TRIM8 may contribute to epilepsy pathogenesis by modulating the stability of key neuronal proteins.

The glucocorticoid receptor, encoded by the NR3C1 gene, has emerged as an important regulator of neuronal function beyond its classical roles in stress response and metabolism [11]. Accumulating evidence suggests that glucocorticoid receptor signaling exerts neuroprotective and anticonvulsant effects [12]. Studies have shown reduced glucocorticoid receptor expression in hippocampal neurons and glial cells of patients with drug-resistant temporal lobe epilepsy, particularly in those comorbid with depression [13]. Furthermore, treatment with the glucocorticoid receptor-specific modulator CORT108297 reduced brain injury after status epilepticus in epileptic mice [14,15]. Mechanistically, glucocorticoid signaling has been shown to regulate AMPAR trafficking and phosphorylation through multiple pathways, including interactions between glucocorticoid receptor-associated complexes and AMPAR regulatory proteins [16,17,18]. Bioinformatic analysis using the UbiBrowser database predicted TRIM8 as a potential E3 ubiquitin ligase targeting NR3C1, prompting us to investigate whether TRIM8 regulates NR3C1 protein stability in the context of epilepsy.

Based on this, we hypothesized that TRIM8 mediates the polyubiquitination of NR3C1, promoting its proteasomal degradation and thereby disinhibiting AMPAR phosphorylation to contribute to epileptogenic pathology. Accordingly, this study employed both in vitro and in vivo epileptic models to explore the molecular regulatory basis and downstream signaling mechanisms by which TRIM8 modulates the NR3C1-AMPAR axis.

## 2. Materials and Methods

Cell Culture and Reagents

The primary mouse hippocampal neuron cells and the cell culture system were purchased from iCell Bioscience Inc. (Shanghai, China). The artificial cerebrospinal fluid was purchased from Leagene Bio (Beijing, China). All cells were maintained at 37 °C in a humidified incubator with 5% CO_2_.

Establishment of the Kainic Acid-Induced Epilepsy Mouse Model

Adult wild-type C57B/L6 mice (8–10 weeks old, 22–25 g) were anesthetized by intraperitoneal injection of pentobarbital sodium. Their heads were then fixed on a stereotactic apparatus, and the hair was shaved and disinfected. The scalp was cut to expose the skull. The injection coordinates of the right hippocampal CA3 region were determined according to the mouse brain atlas (AP −2.0 mm, ML ±1.5 mm, DV −1.8 mm, with Bregma as the reference). After drilling a hole, a microinjection syringe was used to inject KA (2 nmol/μL, volume 0.5 μL, flow rate 0.1 μL/min). The needle was left in place for 5 min to prevent backflow, and the wound was sutured.

Mice were monitored for 24 h postoperatively, and seizure behaviors were scored using the modified Racine scale (Table 1) by investigators blinded to group allocation. Successful KA-induced epileptic modeling was defined as the occurrence of stage III–V acute seizures. During the chronic phase (days 15–30 post-KA injection), spontaneous recurrent seizures (SRSs) were defined as behavioral seizures of at least Racine stage III. The number of SRSs, latency to the first SRS, average seizure duration, Racine stage distribution, and total seizure burden were recorded. Total seizure burden was calculated as the product of SRS frequency and total seizure duration during the chronic phase. On day 30 post-injection, mice were randomly assigned for tissue collection. A total of 6 mice were randomly sacrificed for sample collection: 3 mice per group were anesthetized with 0.3% pentobarbital sodium at 30 mg/kg intraperitoneally, and the mouse brain tissues were fixed by cardiac perfusion for pathological staining; the remaining 3 mice per group were immediately decapitated after intraperitoneal injection of pentobarbital sodium for anesthesia, the scalp and skull were cut open, and the brain tissues were placed on ice. The right hippocampal tissue and temporal lobe tissue were separated for WB detection.

**AAV-Mediated Gene Manipulation In Vivo**

For in vivo knockdown experiments, adeno-associated virus serotype 9 (AAV9) carrying shRNA targeting mouse TRIM8 (AAV9-shRNA-TRIM8) or scrambled control shRNA (AAV9-shRNA-scramble) was stereotaxically injected into the hippocampus two weeks prior to KA administration. Viral vectors were injected bilaterally into the hippocampal CA3 region (1 μL per side, titer: 1 × 10^12^ vg/mL) using the same coordinates described above. Successful transduction was confirmed by eGFP fluorescence in hippocampal sections.

**In Vitro Epileptiform Activity Model**

Primary mouse hippocampal neurons (MIC-iCell-n006, iCell Bioscience Inc., Shanghai, China) were seeded in primary neuron-specific culture medium and cultured for 3 days. On days 4–5 post-seeding, neurons were transfected with OE-TRIM8, OE-NR3C1, or the corresponding empty overexpression control vector (OE-NC) using Lipofectamine 3000 (L3000150, Thermo Fisher Scientific, Waltham, MA, USA) according to the manufacturer’s instructions. OE-NC served as the negative-control vector and did not encode TRIM8 or NR3C1. After 4–6 h of transfection, the transfection mixture was replaced with fresh, pre-warmed complete neuronal culture medium to minimize reagent toxicity. On day 7 post-seeding, epileptiform activity was induced using Mg^2+^-free ACSF.

To induce epileptiform activity, neurons were incubated with Mg^2+^-free ACSF for 3 h, which removes the voltage-dependent Mg^2+^ block of NMDA receptors and induces synchronized neuronal discharges. Control neurons were incubated with normal ACSF containing 1.2 mM MgCl_2_ for the same duration.

The following experimental groups were established: Experiment 1 (to assess the effect of TRIM8 overexpression): Group 1: OE-NC + normal ACSF (OE-NC + Normal), Group 2: OE-TRIM8 + normal ACSF (OE-TRIM8 + Normal), Group 3: OE-NC + Mg^2+^-free ACSF (OE-NC + Model), Group 4: OE-TRIM8 + Mg^2+^-free ACSF (OE-TRIM8 + Model). Experiment 2 (to assess the rescue effect of NR3C1 overexpression): Group 1: OE-NC + normal ACSF (OE-NC + Normal), Group 2: OE-NC + Mg^2+^-free ACSF (OE-NC + Model), Group 3: OE-TRIM8 + Mg^2+^-free ACSF (OE-TRIM8 + Model), Group 4: OE-NR3C1 + Mg^2+^-free ACSF (OE-NR3C1 + Model), Group 5: OE-TRIM8 + OE-NR3C1 + Mg^2+^-free ACSF (OE-TRIM8 + OE-NR3C1 + Model).

**Cell Viability Assay**

Cell viability was assessed using the Cell Counting Kit-8 (CCK-8) assay. Briefly, neurons were seeded into 96-well plates at an appropriate density. Following experimental treatments, the culture medium was aspirated, and 110 μL of CCK-8 working solution (CCK-8 reagent diluted 1:10 in serum-free medium) was added to each well. After incubation at 37 °C for 2 h, absorbance was measured at 450 nm using a microplate reader (ELx800, BioTek Instruments, Winooski, VT, USA). Each group consisted of 4 replicates.

**Co-Immunoprecipitation (Co-IP)**

To assess the interaction between TRIM8 and NR3C1, co-immunoprecipitation was performed. Cell lysates were prepared in IP lysis buffer containing protease inhibitors. Lysates were pre-cleared with Protein A/G agarose beads and then incubated overnight at 4 °C with anti-Flag antibody (TRIM8 1:1000, Abcam, Cambridge, UK; NR3C1 1:5000, Proteintech, Wuhan, Hubei, China) or normal IgG (negative control) (1:2000, Servicebio, Wuhan, Hubei, China). Immune complexes were captured using Protein A/G agarose beads, washed extensively, and eluted by boiling in SDS sample buffer.

The immunoprecipitated proteins were subsequently analyzed by Western blot using antibodies against TRIM8 and NR3C1. To assess the ubiquitination level of NR3C1, primary hippocampal neurons were co-transfected with HA-ubiquitin and either OE-NR3C1 alone or together with OE-TRIM8. Cell lysates were immunoprecipitated with anti-NR3C1 antibody or control IgG, followed by immunoblotting with anti-ubiquitin antibody to detect ubiquitinated NR3C1.

**Protein Stability Assay**

To evaluate NR3C1 protein stability, neurons were transfected with OE-TRIM8 or OE-NC for 16 h and then treated with the protein synthesis inhibitor cycloheximide (CHX; 0.1 μM). Cell lysates were collected at 0, 3, 6, and 9 h post-CHX treatment for Western blot analysis of NR3C1 protein levels. The half-life of NR3C1 protein was determined by plotting the relative protein levels against time.

**Real-Time Quantitative PCR (RT-qPCR)**

Total RNA was isolated using the Total RNA Extraction Kit (Yeasen, Shanghai, China) and subsequently converted into cDNA via the Prime Script RT Reagent Kit (Takara, Beijing, China). RT-qPCR was performed using cDNA as the template, TB Green Premix Ex Taq II (Takara, Beijing, China) as the premix, and the primers shown in Table 2.

**Intracellular Calcium Measurement by Flow Cytometry**

Intracellular calcium levels were measured using the calcium-sensitive fluorescent indicators Fluo-4 AM for cytosolic Ca^2+^ and Rhod-2 AM for mitochondrial Ca^2+^. Primary hippocampal neurons were seeded into 6-well plates at a density of 1 × 10^5^ cells per well. After the indicated experimental treatments, cells were harvested by gentle trypsinization and centrifugation. Cell pellets were resuspended in 300 μL of PBS containing 1 μM Fluo-4 AM or Rhod-2 AM and incubated at 37 °C in the dark for 30 min. After two washes with PBS, cells were resuspended in 300 μL of PBS and immediately analyzed using a flow cytometer (BD Biosciences, Franklin Lakes, NJ, USA).

Flow-cytometry data were analyzed using FlowJo software (Version 10.8). Debris was excluded based on FSC-A versus SSC-A plots, and doublets or cell aggregates were excluded using FSC-A versus FSC-H plots. The mean fluorescence intensity (MFI) of Fluo-4 AM or Rhod-2 AM was calculated from the final gated single-cell population. Representative two-parameter FACS plots showing the gating strategy are provided in Supplementary Figures S1 and S2. Each experiment was performed with three independent biological replicates.

**Electrophysiological recording**

Spontaneous action potentials (APs) were recorded using the whole-cell patch-clamp technique in cultured hippocampal neurons under current-clamp mode, with an extracellular solution containing appropriate ions and a potassium-based intracellular solution. For recording spontaneous excitatory postsynaptic currents (sEPSCs), the intracellular solution was replaced with a cesium-based solution, and bicuculline was added to the extracellular solution to block inhibitory inputs; recordings were performed under voltage-clamp mode at a holding potential of −70 mV. Two experimental conditions were included: a control group (with Mg^2+^) and a Mg^2+^-free epilepsy-like model group, in which neurons were first equilibrated for 30 min in Mg^2+^-containing solution and then incubated in Mg^2+^-free solution for 3 h. Signals were amplified, low-pass filtered, and analyzed for AP frequency and amplitude, as well as sEPSC frequency and amplitude.

**Immunofluorescence staining**

Paraffin-embedded mouse brain sections were deparaffinized, rehydrated, and subjected to microwave-based antigen retrieval in citrate buffer. After endogenous peroxidase activity was blocked with hydrogen peroxide, sections were blocked with 3% BSA and incubated overnight at 4 °C with primary antibodies against NeuN (1:200, Abcam, Cambridge, UK), TRIM8 (1:100, Proteintech, Wuhan, Hubei, China), and NR3C1 (1:100, Huabio, Hangzhou, Zhejiang, China). Sections were then incubated with species-matched HRP-conjugated secondary antibodies, followed by tyramide signal amplification using FITC-, CY3-, or CY5-conjugated tyramide. For multiplex staining, antigen retrieval and blocking were repeated between sequential staining rounds to remove previously bound antibody complexes and reduce signal carryover. Nuclei were counterstained with DAPI, and sections were mounted with anti-fade mounting medium. Fluorescence images were acquired using an Olympus VS200 slide scanner (Olympus, Tokyo, Japan) and analyzed with ImageJ software (Version 1.53, National Institutes of Health, Bethesda, MD, USA).

**Western blot (WB)**

We prepared the cell protein extract, separated it by SDS-PAGE electrophoresis (Biosharp, Shanghai, China), and then transferred it to a PVDF membrane (Sigma Aldrich, Shanghai, China). After blocking, the PVDF membrane was incubated overnight at 4 °C with primary antibodies (TRIM8 1:1000, Abcam, Cambridge, UK; β-actin 1:50,000, Abclonal, Wuhan, Hubei, China; GluR1 1:2000, Thermo Fisher Scientific, Waltham, MA, USA; GluR2 1:2000, Huabio, Hangzhou, Zhejiang, China; p-GluR1 (s831) 1:1000, Huabio, Hangzhou, Zhejiang, China; p-GluR2 (s880) 1:2000, Abclonal, Wuhan, Hubei, China; NR3C1 1:5000, Proteintech, Wuhan, Hubei, China). The membranes were washed with PBS three times, and incubated with goat anti-rabbit IgG (H + L) secondary antibody (1:8000, Abclonal, Wuhan, Hubei, China) for 2 h at room temperature. The bands were subsequently detected with Torchlight’s Hypersensitive ECL Western HRP Substrate (zen-bio, Shanghai, China) and captured using the Fluorescence Image Analysis System Software V2.0 (Tanon, Shanghai, China).

**Statistical analysis**

Data are presented as mean ± standard deviation (SD). Statistical evaluations were performed using SPSS 20.0 software (IBM, New York, NY, USA). To compare data across groups, one-way analysis of variance (ANOVA) was employed. In cases where the variance was homogeneous, the LSD test was utilized for post hoc comparisons. Conversely, when the variance was not homogeneous, Tamhane’s T2 test was applied. A two-tailed *p* < 0.05 was considered significant.

## 3. Results

### 3.1. Expression and Localization of TRIM8 Protein in the Brain Tissues of Epileptic Mice

**Figure 1:**
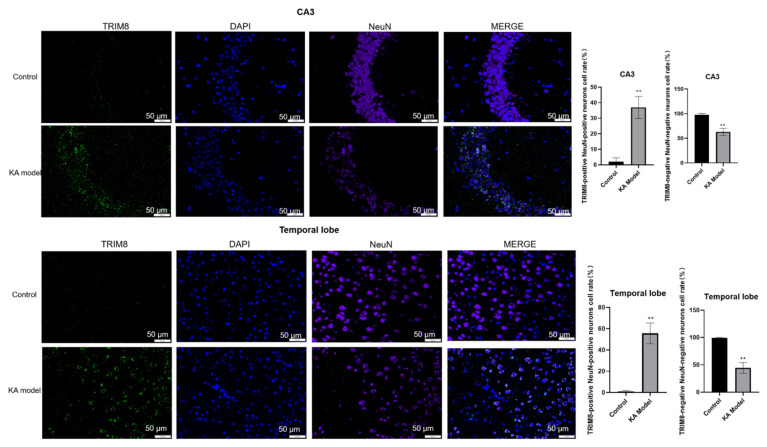
Immunofluorescence double staining of NeuN (purple) and TRIM8 (green) was performed to assess TRIM8 expression in NeuN-positive neurons in the right hippocampal CA3 region and temporal lobe of control and KA-induced epileptic mice. TRIM8-positive and TRIM8-negative neurons were quantified among total NeuN-positive cells (*n* = 3). Data are presented as mean ± SD. Compared with the Control. ** *p* < 0.01.

To investigate the expression and cellular localization of TRIM8 in KA-induced epileptic mice, immunofluorescence double staining was performed on brain sections from the right hippocampus and temporal lobe regions. Double staining for NeuN (neuronal marker) and TRIM8 was conducted to assess TRIM8 expression in neurons (Figure 1). Statistical analysis of TRIM8-positive and -negative cell rates showed that in the CA3 region of the hippocampus, compared with the control group, the epilepsy model group exhibited a significantly increased TRIM8-positive cell rate (*p* < 0.01) and a significantly decreased TRIM8-negative cell rate (*p* < 0.01). In the temporal lobe, similarly, the TRIM8-positive cell rate was markedly higher in the model group (*p* < 0.01), while the TRIM8-negative cell rate was significantly lower (*p* < 0.01). Thus, in KA-induced epileptic mice, TRIM8 expression is markedly increased in the hippocampal CA3 region and the temporal lobe.

### 3.2. Correlation Between TRIM8 Expression and Glucocorticoid Receptor NR3C1 Expression in the Brain of Epileptic Mice

**Figure 2:**
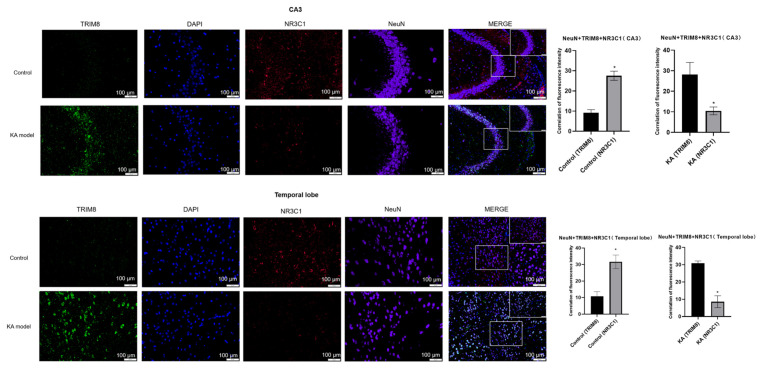
Immunofluorescence triple staining for NeuN (purple), TRIM8 (green), and NR3C1 (red) was performed to observe the expression patterns of TRIM8 and NR3C1 in the right hippocampal CA3 region and temporal lobe, with NeuN staining used to indicate the neuronal layer/regions (*n* = 3). The white boxes in the merged images indicate the selected regions shown at higher magnification in the inset panels. “*” represents a significant correlation at the 0.05 level (two-tailed).

To investigate the relationship between TRIM8 and the glucocorticoid receptor NR3C1 in epileptic mice, immunofluorescence triple staining for NeuN (neuronal marker), TRIM8, and NR3C1 was performed on brain sections from the right hippocampus and temporal lobe regions (Figure 2). DAPI was used to stain nuclei (blue). TRIM8 immunoreactivity (green) was predominantly localized in the cytoplasm, NR3C1 immunoreactivity (red) was also mainly cytoplasmic, and NeuN (purple) was primarily nuclear. Quantitative analysis of fluorescence intensity revealed a significant negative correlation between TRIM8 and NR3C1 expression in the control group (*p* = 0.028). Similarly, in the epilepsy model group, a significant negative correlation between TRIM8 and NR3C1 expression was also observed (*p* = 0.040). These results demonstrate that an inverse relationship between TRIM8 and NR3C1 immunoreactivity in the CA3 neuronal layer and temporal lobe regions of both control and epileptic mice, suggesting in vivo evidence for a potential regulatory relationship between these two proteins.

### 3.3. Overexpression of TRIM8 Enhances AMPA Receptor Phosphorylation and Reduces NR3C1 Expression In Vitro Epileptiform Model

**Figure 3:**
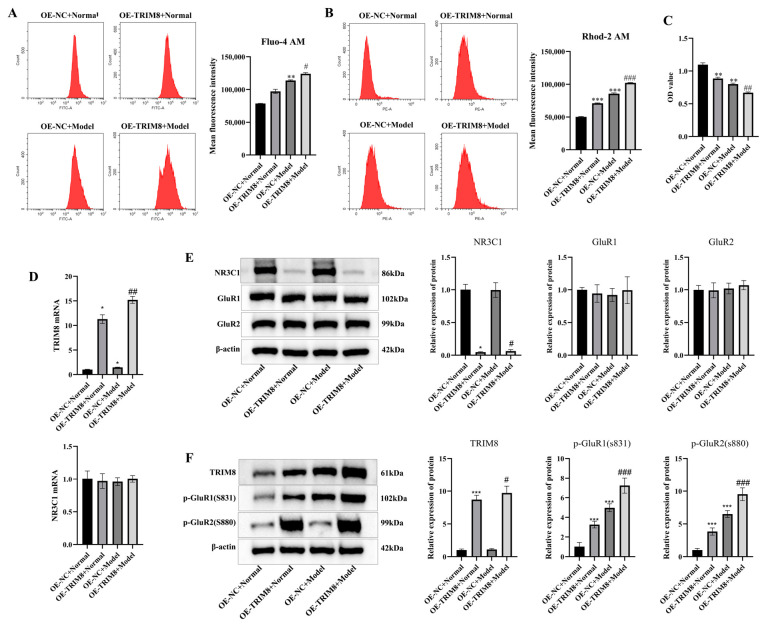
Overexpression of TRIM8 enhances AMPA receptor-mediated synaptic excitability and reduces NR3C1 expression in the epileptic cell model (*n* = 3). (**A**,**B**) Flow cytometry was used to analyze the fluctuations in intracellular calcium ion concentration; (**C**) Cell viability was detected by CCK8; (**D**) Changes in mRNA and protein expression of TRIM8 and NR3C1; (**E**,**F**) Protein banding pattern diagram; changes in protein expression of GluR1 and GluR2, p-GluR1 (S831) and p-GluR2 (S880). Compared with the OE-NC + Normal, * *p* < 0.05, ** *p* < 0.01, *** *p* < 0.001; compared with the OE-NC + Model, ^#^ *p* < 0.05, ^##^ *p* < 0.01, ^###^ *p* < 0.001.

The in vitro epileptiform activity model was established by incubating mouse primary hippocampal neurons with Mg^2+^-free ACSF. Neurons were transfected with OE-TRIM8 or the empty overexpression control vector (OE-NC) prior to model induction. Flow cytometry analysis revealed that cytosolic calcium levels (Fluo-4 AM) and mitochondrial calcium levels (Rhod-2 AM) increased significantly following epileptiform model treatment (*p* < 0.01), and overexpression of TRIM8 further enhanced this increase (*p* < 0.05) (Figure 3A,B and Supplementary Figure S1). CCK-8 assay results showed that compared to the OE-NC + Normal group, cell viability was modestly decreased in both the OE-TRIM8 + Normal group and the OE-NC + Model group (*p* < 0.001). Under Mg^2+^-free conditions, the viability in the OE-TRIM8 + Model group was further decreased compared to the OE-NC + Model group (*p* < 0.001) (Figure 3C). PCR results indicated that TRIM8 mRNA was significantly upregulated in all OE-TRIM8 groups (*p* < 0.05 or *p* < 0.01), while NR3C1 mRNA showed no significant changes across groups (Figure 3D). Western blot analysis demonstrated that TRIM8 protein levels were significantly elevated in all OE-TRIM8 groups (*p* < 0.05 or *p* < 0.001), whereas NR3C1 protein levels were significantly reduced (*p* < 0.05) (Figure 3D,E). Notably, while NR3C1 mRNA levels remained unchanged, NR3C1 protein levels were significantly decreased in TRIM8-overexpressing neurons, suggesting post-transcriptional regulation of NR3C1 by TRIM8. Total protein levels of GluR1 and GluR2 did not change significantly; however, their phosphorylated forms, p-GluR1 (S831) and p-GluR2 (S880), were significantly increased in both OE-TRIM8 groups and Model groups (*p* < 0.001), with the highest expression observed in the OE-TRIM8 + Model group (Figure 3E,F).

### 3.4. TRIM8 Interacts with NR3C1 and Promotes Its Ubiquitination and Proteasomal Degradation in Neurons

**Figure 4:**
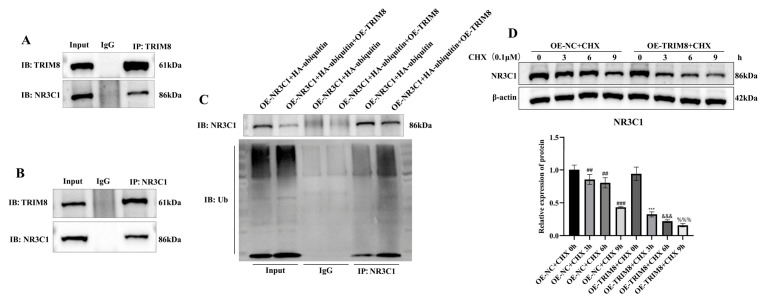
TRIM8 Regulates the Binding and Ubiquitination of NR3C1 in Mouse Hippocampal Neurons. (**A**) Expression of TRIM8 and NR3C1 in protein samples after co-IP with NR3C1 antibody, detected by WB. (**B**) Expression of TRIM8 and NR3C1 in protein samples after co-IP with TRIM8 antibody, detected by WB. (**C**) The enrichment of NR3C1 protein after IP (NR3C1) was detected by WB. The ubiquitination expression in the protein samples after IP (NR3C1) was detected by WB. (**D**) Changes in NR3C1 protein expression (*n* = 3). Compared with the OE-NC + CHX 0 h group, ^##^ *p* < 0.01, ^###^ *p* < 0.001; compared with the OE-NC + CHX 3 h group, *** *p* < 0.001; compared with the OE-NC + CHX 6 h group, ^&&&^ *p* < 0.001; compared with the OE-NC + CHX 9 h group, ^%%%^ *p* < 0.001.

Co-immunoprecipitation (Co-IP) was performed to assess the interaction between TRIM8 and NR3C1. Western blot analysis was performed to detect the expression of TRIM8 and NR3C1 in the immunoprecipitated protein samples. As shown in Figure 4A, distinct protein bands were detected in the IP group, confirming the successful enrichment of NR3C1 protein. Notably, TRIM8 protein was also co-precipitated with NR3C1, indicating an association between the two proteins. Similarly, results from Figure 4B demonstrate successful enrichment of TRIM8 protein, with NR3C1 detected in the precipitate, further supporting TRIM8 and NR3C1 may be part of a complex.

To further investigate the effect of TRIM8 on NR3C1 ubiquitination, neurons were co-transfected with OE-NR3C1 and HA-ubiquitin, with or without OE-TRIM8. After immunoprecipitation with anti-NR3C1 antibody, Western blot analysis confirmed successful enrichment of NR3C1 protein in both groups (Figure 4C). Importantly, ubiquitination assay revealed that the polyubiquitination level of NR3C1 was markedly higher in the OE-TRIM8 co-transfected group compared to the control group (Figure 4C), providing direct evidence that TRIM8 promotes NR3C1 ubiquitination.

To evaluate the effect of TRIM8 on NR3C1 protein stability, neurons were transfected with OE-TRIM8 or OE-NC for 16 h and then treated with the protein synthesis inhibitor cycloheximide (CHX; 0.1 μM)**.** Samples were collected at 0, 3, 6, and 9 h for Western blot analysis (Figure 4D). The results showed that in the OE-NC control group, the NR3C1 protein level decreased in a time-dependent manner with prolonged CHX treatment: compared to the 0-h time point, protein expression was significantly reduced at both 3 and 6 h (*p* < 0.01) and further decreased by 9 h (*p* < 0.001). Overexpression of TRIM8 markedly accelerated this degradation process: at the same time points of CHX treatment (3, 6, and 9 h), the NR3C1 protein levels in the OE-TRIM8 group decreased more rapidly compared to the OE-NC group, with differences reaching statistical significance (*p* < 0.001). Furthermore, at the 0-h time point of CHX treatment, there was no significant difference in the basal NR3C1 protein levels between the OE-TRIM8 and OE-NC groups. These results indicate that overexpression of TRIM8 significantly reduces the stability of the NR3C1 protein and promotes its proteasomal degradation.

### 3.5. NR3C1 Overexpression Rescues TRIM8-Induced Calcium Dysregulation and AMPAR Phosphorylation

**Figure 5:**
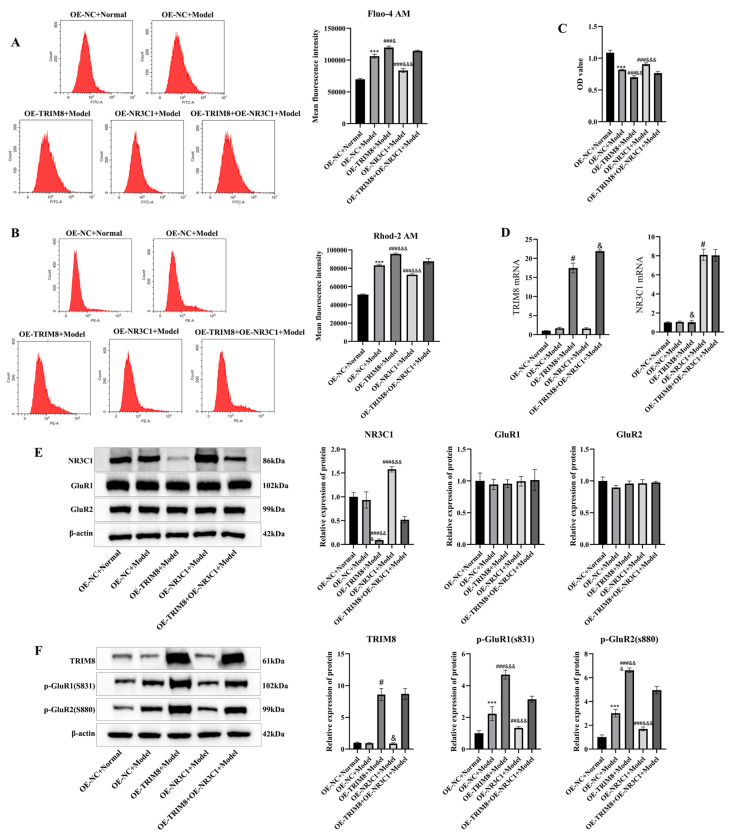
Overexpression of TRIM8 exacerbates neuronal injury in an epilepsy model by targeting NR3C1 to promote AMPAR phosphorylation (*n* = 3). (**A**,**B**) Flow cytometry was used to analyze the fluctuations in intracellular calcium ion concentration. (**C**) Cell viability was detected by CCK8. (**D**) Changes in mRNA expression of TRIM8 and NR3C1. (**E**,**F**) Changes in protein expression of TRIM8, NR3C1, GluR1, GluR2, p-GluR1 (S831) and p-GluR2 (S880). Compared with the OE-NC + Normal, *** *p* < 0.001; compared with the OE-NC + Model, ^#^ *p* < 0.05, ^##^ *p* < 0.01, ^###^ *p* < 0.001; compared with the OE-TRIM8 + OE-NR3C1 + Model, ^&^ *p* < 0.05, ^&&^ *p* < 0.01, ^&&&^ *p* < 0.001.

To further validate that TRIM8 regulates AMPAR phosphorylation through NR3C1, and to assess the protective effect of NR3C1 in the Mg^2+^-free ACSF-induced epileptiform model, rescue experiments were conducted. Flow cytometry analysis with Fluo-4 AM and Rhod-2 AM for evaluating intracellular calcium dynamics revealed consistent patterns: intracellular calcium levels were significantly elevated under model conditions (*p* < 0.001), further exacerbated by OE-TRIM8, but attenuated by OE-NR3C1 (both *p* < 0.001). Co-overexpression of TRIM8 and NR3C1 reversed the effects of TRIM8 overexpression alone (Figure 5A,B and Supplementary Figure S2). CCK-8 assays revealed that cell viability was significantly reduced in the Model group compared to the Normal group (*p* < 0.001). Notably, overexpression of TRIM8 further exacerbated this reduction in viability under model conditions, whereas overexpression of NR3C1 significantly attenuated it (both *p* < 0.001). Furthermore, the decreased viability caused by OE-TRIM8 was substantially rescued by co-overexpression of NR3C1 (Figure 5C). PCR analysis showed no significant change in NR3C1 mRNA levels induced by model treatment. However, OE-NR3C1 successfully elevated its mRNA levels, and this elevation was attenuated when co-expressed with TRIM8. TRIM8 mRNA levels were specifically increased in the OE-TRIM8 group and when NR3C1 was co-overexpressed (Figure 5D).

Western blot analysis of protein levels provided key mechanistic insights (Figure 5E,F). While total GluR1 and GluR2 protein levels remained unchanged across groups, the phosphorylation levels of GluR1 at Ser831 and GluR2 at Ser880 were markedly increased under model conditions (*p* < 0.001). This increase was further enhanced by OE-TRIM8 but was significantly suppressed by OE-NR3C1 (*p* < 0.001). Co-overexpression partially neutralized these effects. Concurrently, NR3C1 protein expression was significantly decreased by OE-TRIM8 and increased by OE-NR3C1 under epileptiform conditions (*p* < 0.001). TRIM8 protein was confirmed to be overexpressed in the corresponding group. These results collectively indicate that in the Mg^2+^-free ACSF-induced epileptiform model, TRIM8 promotes neuronal damage and enhances AMPAR phosphorylation (evidenced by increased subunit phosphorylation) by mediating NR3C1 ubiquitination and accelerating its proteasomal degradation, while NR3C1 overexpression exerts a protective effect that counteracts TRIM8-induced changes.

### 3.6. In Vivo Validation of the Knockdown Effect of AAV9-shRNA-TRIM8 and Its Influence on the NR3C1-AMPAR Axis

To validate the regulatory role of TRIM8 on the seizure behavior and the NR3C1-AMPAR axis in vivo, AAV9-shRNA-TRIM8 and its control virus were stereotaxically injected into the mouse hippocampus two weeks prior to KA administration. Stereotaxic injection of AAV9-shRNA-TRIM8 produced detectable eGFP expression in the hippocampal injection region, confirming successful viral transduction. Subsequent Western blot and PCR analyses revealed that, compared to the control group, the mRNA levels of TRIM8 in the hippocampal tissue of the AAV9-shRNA-TRIM8 group were significantly reduced (*p* < 0.01), and the protein levels were also markedly decreased (*p* < 0.001), confirming the successful knockdown of TRIM8 (Supplementary Figure S3).

**Figure 6:**
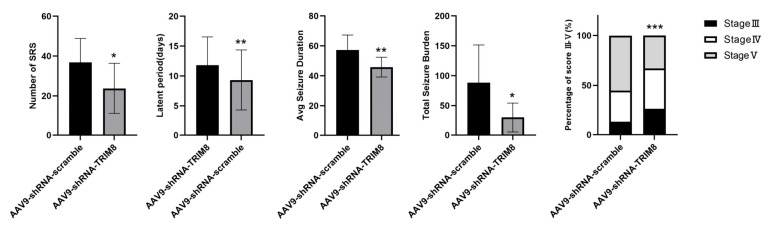
shTRIM8 knockdown mitigates seizure severity in the KA-induced epilepsy mouse model (*n* = 6). Compared with the scramble control by unpaired *t*-test or Mann–Whitney U test, * *p* < 0.05, ** *p* < 0.01, *** *p* < 0.001.

In behavioral assessments, compared with the AAV9-shRNA-scramble control group, the AAV9-shRNA-shTRIM8 group exhibited a significant reduction in seizure frequency between days 15–30 post-KA injection (*p* < 0.05), a prolonged latency to the first Stage III seizure (*p* < 0.01), and a shorter average seizure duration (*p* < 0.01). Total seizure burden was also markedly decreased (*p* < 0.05). Additionally, the distribution of seizure severity shifted toward less severe stages in the shTRIM8 group, with a lower proportion of Stage V seizures and a higher proportion of Stage III seizures (*p* < 0.001) (Figure 6). These behavioral findings demonstrate that TRIM8 knockdown attenuates KA-induced seizure severity in vivo.

Under Mg^2+^-free conditions, the frequency of spontaneous APs and the amplitude of sEPSCs in hippocampal neurons were significantly increased compared to conditions with Mg^2+^, indicating that Mg^2+^ withdrawal successfully induced neuronal network hyperexcitability and enhanced postsynaptic excitatory input.

Under control conditions with Mg^2+^, there were no significant differences in the amplitude or frequency of spontaneous APs between the OE-TRIM8 group and the control group, suggesting that TRIM8 overexpression does not significantly alter AP generation mechanisms or sodium channel-mediated depolarization under basal conditions. Under Mg^2+^-free conditions, the frequency of spontaneous APs in the OE-TRIM8 group was significantly higher than that in the control group, whereas the AP amplitude remained unchanged. This indicates that TRIM8 overexpression markedly amplifies neuronal firing under epileptiform conditions without affecting the intrinsic electrophysiological properties of individual APs.

**Figure 7:**
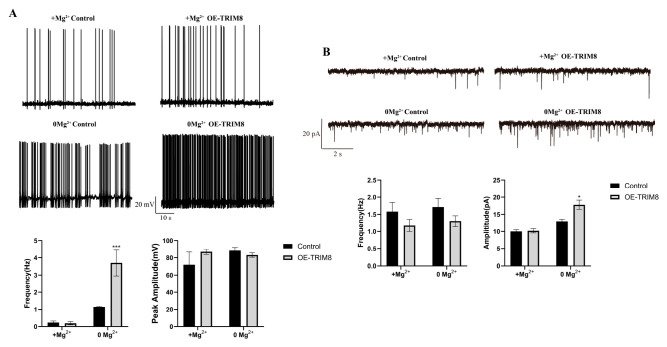
Effects of TRIM8 overexpression on spontaneous APs and sEPSCs under control and Mg^2+^-free conditions in hippocampal neurons. (**A**) Summary bar graphs showing the spontaneous APs in control and OE-TRIM8 neurons under conditions with Mg^2+^ (+Mg^2+^) and without Mg^2+^ (0 Mg^2+^). (**B**) Summary bar graphs showing the sEPSCs in control and OE-TRIM8 neurons under +Mg^2+^ and 0 Mg^2+^ conditions. *n* = 8 neurons from 5 mice per group. Compared with the Mg^2+^-free control group, * *p* < 0.05, *** *p* < 0.001.

Under conditions with Mg^2+^, there were no significant differences in sEPSC amplitude or frequency between the OE-TRIM8 group and the control group, suggesting that TRIM8 overexpression does not significantly affect excitatory synaptic transmission under basal conditions. Under Mg^2+^-free conditions, the amplitude of sEPSCs in the OE-TRIM8 group was significantly higher than that in the control group, while the sEPSC frequency showed no significant change. This suggests that TRIM8 primarily enhances postsynaptic AMPA receptor-mediated current strength, rather than altering presynaptic glutamate release (Figure 7).

Triple immunofluorescence staining for NeuN, TRIM8, and NR3C1 was conducted to evaluate their expression in the CA3 region of the right hippocampus and the temporal lobe (Supplementary Figure S4). Compared to the AAV9-shRNA-scramble control group, the AAV9-shRNA-scramble + KA group exhibited a marked increase in TRIM8 fluorescence intensity but a significant decrease in NR3C1 fluorescence intensity in NeuN^+^ neurons, indicating opposite regulation of these proteins following KA-induced excitotoxicity. Following TRIM8 knock-down, TRIM8 fluorescence intensity was reduced, while NR3C1 fluorescence intensity was restored in NeuN^+^ neurons. These results indicate that TRIM8 negatively regulates NR3C1 expression in hippocampal neurons during epileptogenesis.

Further analysis of protein expression in hippocampal tissue (Supplementary Figure S5) showed that KA-induced epilepsy did not cause significant changes in the total protein levels of GluR1 and GluR2, nor did knockdown of TRIM8 significantly alter their expression levels. However, compared with the AAV9-shRNA-scramble group, the expression level of NR3C1 protein in the hippocampal tissue of the KA-induced epilepsy model group was markedly reduced (*p* < 0.001). In contrast, TRIM8 knockdown significantly reversed this reduction, leading to a recovery of NR3C1 protein levels (*p* < 0.01). Additionally, KA-induced epilepsy markedly increased the protein expression levels of p-GluR1 (S831) and p-GluR2 (S880) as well as their ratios to the corresponding total proteins (*p* < 0.001). Compared with the KA-induced epilepsy model group, TRIM8 knockdown significantly reduced the expression of p-GluR1 (S831) (*p* < 0.001) and decreased the expression of p-GluR2 (S880) and its ratio to total protein (*p* < 0.01).

These results indicate that in the KA-induced epilepsy mouse model, TRIM8 knockdown effectively inhibits the decrease in NR3C1 protein caused by epilepsy modeling and blocks the excessive phosphorylation of specific serine sites on the AMPAR subunits GluR1 and GluR2. These in vivo results are consistent with the in vitro findings and support the conclusion that TRIM8 promotes NR3C1 degradation, thereby enhancing AMPAR phosphorylation. The TRIM8-NR3C1-AMPAR axis represents a molecular pathway contributing to seizure susceptibility in the KA-induced epilepsy model.

## 4. Discussion

The present study identifies TRIM8 as a critical regulator of NR3C1 protein stability and downstream AMPAR phosphorylation in epilepsy. We demonstrate that TRIM8 expression is significantly upregulated in neurons of the hippocampal CA3 and the temporal lobe in epileptic mice (Figure 1). Given the established role of the hippocampus, particularly the CA3 subregion, in seizure generation and propagation, the upregulation of TRIM8 in this area is particularly noteworthy [19]. The upregulation of TRIM8 in both neurons and glial cells suggests its involvement in both cell-autonomous and non-cell-autonomous mechanisms of epileptogenesis, potentially linking molecular alterations in neuronal signaling with neuroinflammatory responses [20].

A key finding of our study is the negative correlation between TRIM8 and NR3C1 expression in neurons of both control and epileptic mice (Figure 2). This inverse relationship provides in vivo evidence linking TRIM8 to glucocorticoid receptor signaling in epilepsy. NR3C1, the gene encoding the glucocorticoid receptor, has been increasingly recognized for its neuroprotective functions beyond classical stress responses [11]. Previous studies have demonstrated reduced glucocorticoid receptor expression in hippocampal neurons of patients with drug-resistant temporal lobe epilepsy, particularly those with comorbid depression [13]. Our findings extend this observation by identifying TRIM8 as a potential upstream regulator of NR3C1 protein stability in epilepsy. The interaction between TRIM8 and NR3C1, confirmed by co-immunoprecipitation (Figure 4A,B), together with the direct demonstration that TRIM8 overexpression significantly enhanced NR3C1 polyubiquitination (Figure 4C), establishes that TRIM8 functions as an E3 ubiquitin ligase promoting NR3C1 proteasomal degradation. This interpretation is supported by our cycloheximide chase experiments demonstrating that TRIM8 overexpression significantly accelerates NR3C1 protein turnover (Figure 4D).

The functional consequences of TRIM8-mediated NR3C1 degradation are manifested through enhanced AMPA receptor phosphorylation. Our results demonstrate that TRIM8 overexpression significantly increases phosphorylation of GluR1 at Ser831 and GluR2 at Ser880 in the Mg^2+^-free ACSF-induced epileptiform model (Figure 3E,F and Figure 5E,F), without altering total GluR1 or GluR2 protein levels. Importantly, AMPAR phosphorylation at these specific sites represents well-established molecular signatures associated with neuronal hyperexcitability in epilepsy models. Ser831 phosphorylation of GluR1 by CaMKII has been shown to increase single-channel conductance [21], while Ser880 phosphorylation of GluR2 by PKC promotes GluR2 internalization and facilitates the incorporation of calcium-permeable AMPA receptors [22]. These phosphorylation changes have been consistently observed in multiple experimental epilepsy models and are considered reliable biochemical indicators of enhanced AMPAR function [23]. The critical role of NR3C1 in counteracting TRIM8 effects was confirmed by rescue experiments, where NR3C1 overexpression attenuated TRIM8-induced calcium dysregulation and cell death (Figure 5). These findings align with recent reports demonstrating that glucocorticoid receptor activation exerts anticonvulsant effects through multiple mechanisms, including suppression of neuroinflammation and modulation of ion channel function [14,24]. Specifically, glucocorticoid signaling has been shown to directly regulate AMPAR trafficking and phosphorylation through corticosteroid receptor-dependent mechanisms, including interactions with NSF/GluR2 complexes and SGK-mediated regulation of AMPAR recycling [16,18,25]. Thus, TRIM8-mediated NR3C1 degradation may directly disinhibit these regulatory pathways, leading to enhanced AMPAR phosphorylation.

In vivo validation using AAV9-mediated TRIM8 knockdown in the KA-induced epilepsy model provided compelling evidence for the translational relevance of our findings. Stereotaxic injection of AAV9-shRNA-TRIM8 resulted in hippocampal transduction (Supplementary Figure S3A) and significant TRIM8 knockdown (Supplementary Figure S3B). Importantly, TRIM8 knockdown attenuated KA-induced seizure severity, as evidenced by reduced seizure frequency, prolonged latency to the first Stage III seizure, and decreased total seizure burden (Figure 6). At the molecular level, TRIM8 knockdown effectively reversed epilepsy-induced NR3C1 reduction and suppressed aberrant AMPAR phosphorylation (Supplementary Figure S5). These behavioral and biochemical findings collectively support the functional significance of the TRIM8-NR3C1-AMPAR axis in epileptogenesis and identify this pathway as a potential therapeutic target.

To further dissect the functional impact of TRIM8 on neuronal excitability, we performed whole-cell patch-clamp recordings in primary hippocampal neurons (Figure 7). Under control conditions (with Mg^2+^), TRIM8 overexpression did not alter the frequency or amplitude of sAPs or sEPSCs, suggesting that TRIM8 does not affect basal neuronal firing or synaptic transmission. However, under Mg^2+^-free epileptiform conditions, TRIM8 overexpression significantly increased the frequency of APs and the amplitude of sEPSCs. This pattern indicates that TRIM8 primarily enhances postsynaptic AMPAR-mediated current strength by increasing the function or expression of postsynaptic AMPA receptors. These electrophysiological findings complement our biochemical data showing enhanced AMPAR phosphorylation, and collectively demonstrate that TRIM8 drives network hyperexcitability by potentiating postsynaptic receptor function.

In addition, triple immunofluorescence staining for NeuN, TRIM8, and NR3C1 in the right hippocampus following AAV9-mediated TRIM8 knockdown provided further in vivo evidence for the regulatory relationship between TRIM8 and NR3C1 (Supplementary Figure S4). Consistent with the Western blot results (Supplementary Figure S5), KA-induced epilepsy led to a marked increase in TRIM8 fluorescence intensity in the hippocampal neuronal layer, whereas NR3C1 fluorescence intensity was significantly decreased. This inverse change aligns with our earlier correlation analysis (Figure 2) and supports the conclusion that TRIM8 upregulation is associated with reduced NR3C1 expression in hippocampal tissue. Notably, TRIM8 knockdown significantly attenuated the KA-induced increase in TRIM8 and concurrently restored NR3C1 immunoreactivity, further confirming that TRIM8 negatively regulates NR3C1 protein levels in vivo. These results support an inverse association between TRIM8 and NR3C1 immunoreactivity during epileptogenesis, with TRIM8 knockdown reversing the pathological downregulation of NR3C1.

Several limitations of this study should be acknowledged. First, the specific lysine residues on NR3C1 targeted by TRIM8 remain to be mapped, and potential regulation by other E3 ligases or deubiquitinating enzymes in this pathway is currently unknown. In addition, although NR3C1 was identified as a functionally relevant substrate of TRIM8 in this study, the broader TRIM8-regulated substrate network during epileptogenesis remains to be further defined. Second, while we focused on AMPAR phosphorylation as a key downstream outcome, other NR3C1-dependent pathways may also contribute to the observed phenotypes. Future studies clarifying additional TRIM8-dependent targets and signaling events may provide a more comprehensive understanding of how TRIM8 regulates neuronal excitability in epilepsy.

## 5. Conclusions

In conclusion, our study identifies TRIM8 as a novel regulator of NR3C1 protein stability in epilepsy. By promoting NR3C1 degradation, TRIM8 disinhibits downstream pathways that regulate AMPAR phosphorylation, a molecular signature associated with neuronal hyperexcitability. The TRIM8-NR3C1-AMPAR axis represents a previously unrecognized regulatory pathway in epileptogenesis and may offer a potential target for therapeutic intervention. Future studies exploring small-molecule inhibitors of TRIM8 or glucocorticoid receptor modulators in preclinical epilepsy models are warranted.

## Figures and Tables

**Table 1:** Behavioral severity scoring criteria.

Stage	Behavioral Manifestation
0	Normal activity, no response.
I	Facial automatism (eye closure, twitching of vibrissae, rhythmical chewing).
II	Head nodding (rhythmical head clonus) in addition to stage I behaviors.
III	Unilateral forelimb clonus in addition to stage II behaviors.
IV	Rearing in addition to stage III behaviors.
V	Generalized tonic–clonic seizure with loss of postural control.

**Table 2:** Primer sequences.

Gene	Primer Sequence-F	Primer Sequence-R
β-actin	CTACCTCATGAAGATCCTGACC	CACAGCTTCTCTTTGATGTCAC
TRIM8	GGAGGAGAGAGAGCAGGACA	TGTCTGCCGCAAGTCTTCAT
NR3C1	TGAAGCTTCGGGATGCCATT	GCTGTCCTTCCACTGCTCTT

## Data Availability

The data involved in this study can be obtained from the corresponding author of this article upon request.

## Supplementary Materials

The following supporting information can be downloaded at: https://www.mdpi.com/article/10.3390/biomedicines14071425/s1, Figure S1: Representative two-parameter FACS plots showing the gating strategy for intracellular calcium analysis in the TRIM8-overexpression experiment. Fluo-4 AM was used to detect cytosolic Ca^2+^, and Rhod-2 AM was used to detect mitochondrial Ca^2+^. Debris was excluded based on FSC-A versus SSC-A plots, and doublets or cell aggregates were excluded using FSC-A versus FSC-H plots. The mean fluorescence intensity (MFI) was calculated from the final gated single-cell population; Figure S2: Representative two-parameter FACS plots showing the gating strategy for intracellular calcium analysis in the NR3C1 rescue experiment. Fluo-4 AM was used to detect cytosolic Ca^2+^, and Rhod-2 AM was used to detect mitochondrial Ca^2+^. Debris was excluded based on FSC-A versus SSC-A plots, and doublets or cell aggregates were excluded using FSC-A versus FSC-H plots. The mean fluorescence intensity (MFI) was calculated from the final gated single-cell population; Figure S3: Expression and localization of TRIM8 protein in hippocampal tissues of epileptic mice. (A) The expression of eGFP in brain tissue; (B) Expression of TRIM8 mRNA and protein in the right hippocampal tissues (*n* = 3). Compared with the Control group, * *p* < 0.05, ** *p* < 0.01, *** *p* < 0.001; compared with the AAV9-shRNA-scramble group, ^#^ *p* < 0.05, ^##^ *p* < 0.01, ^###^ *p* < 0.001; Figure S4: Triple immunofluorescence staining for NeuN, TRIM8, and NR3C1 was performed to evaluate the expression of TRIM8 and NR3C1 in NeuN^+^ neurons in the CA3 region of the right hippocampus and the temporal lobe (*n* = 3). Compared with the AAV9-shRNA-scramble group, * *p* < 0.05, ** *p* < 0.01, *** *p* < 0.001; compared with the AAV9-shRNA-scramble + KA group, ^#^ *p* < 0.05, ^##^ *p* < 0.01, ^###^ *p* < 0.001; Figure S5: The expression of TRIM8, NR3C1, GluR1, p-GluR1 (S831), GluR2, and p-GluR2 (S880) in the right hippocampus tissue (*n* = 3). Compared with the AAV9-shRNA-scramble group, * *p* < 0.05, ** *p* < 0.01, *** *p* < 0.001; compared with the AAV9-shRNA-scramble + KA group, ^#^ *p* < 0.05, ^##^ *p* < 0.01, ^###^ *p* < 0.001.

## Abbreviations

The following abbreviations are used in this manuscript:

AAV9	adeno-associated virus serotype 9
ACSF	artificial cerebrospinal fluid
AMPA	α-amino-3-hydroxy-5-methyl-4-isoxazolepropionic acid
AMPAR	AMPA receptor
APs	action potentials
CA3	cornu ammonis 3
CCK-8	cell counting kit-8
CHX	cycloheximide
Co-IP	co-immunoprecipitation
DG	dentate gyrus
DIV	days in vitro
sEPSCs	excitatory postsynaptic currents
eGFP	enhanced green fluorescent protein
KA	kainic acid
MOI	multiplicity of infection
NeuN	neuronal nuclei
NMDA	N-methyl-D-aspartate
NR3C1	nuclear receptor subfamily 3 group C member 1
OE	overexpression
RT-qPCR	real-time quantitative polymerase chain reaction
shRNA	short hairpin RNA
SRS	spontaneous recurrent seizures
TRIM8	tripartite motif-containing 8
