# Supplementary material for: TRIM8 Promotes Epileptiform Activity by Destabilizing the Glucocorticoid Receptor NR3C1 and Enhancing AMPA Receptor Phosphorylation
Source: Biomedicines. 2026 Jun 24;14(7):1425. doi: 10.3390/biomedicines14071425 (PMC13405928; doi:10.3390/biomedicines14071425)
Supplement: Supplementary file 1 [file biomedicines-14-01425-s001.zip › biomedicines-4266156-supplementary.pdf]

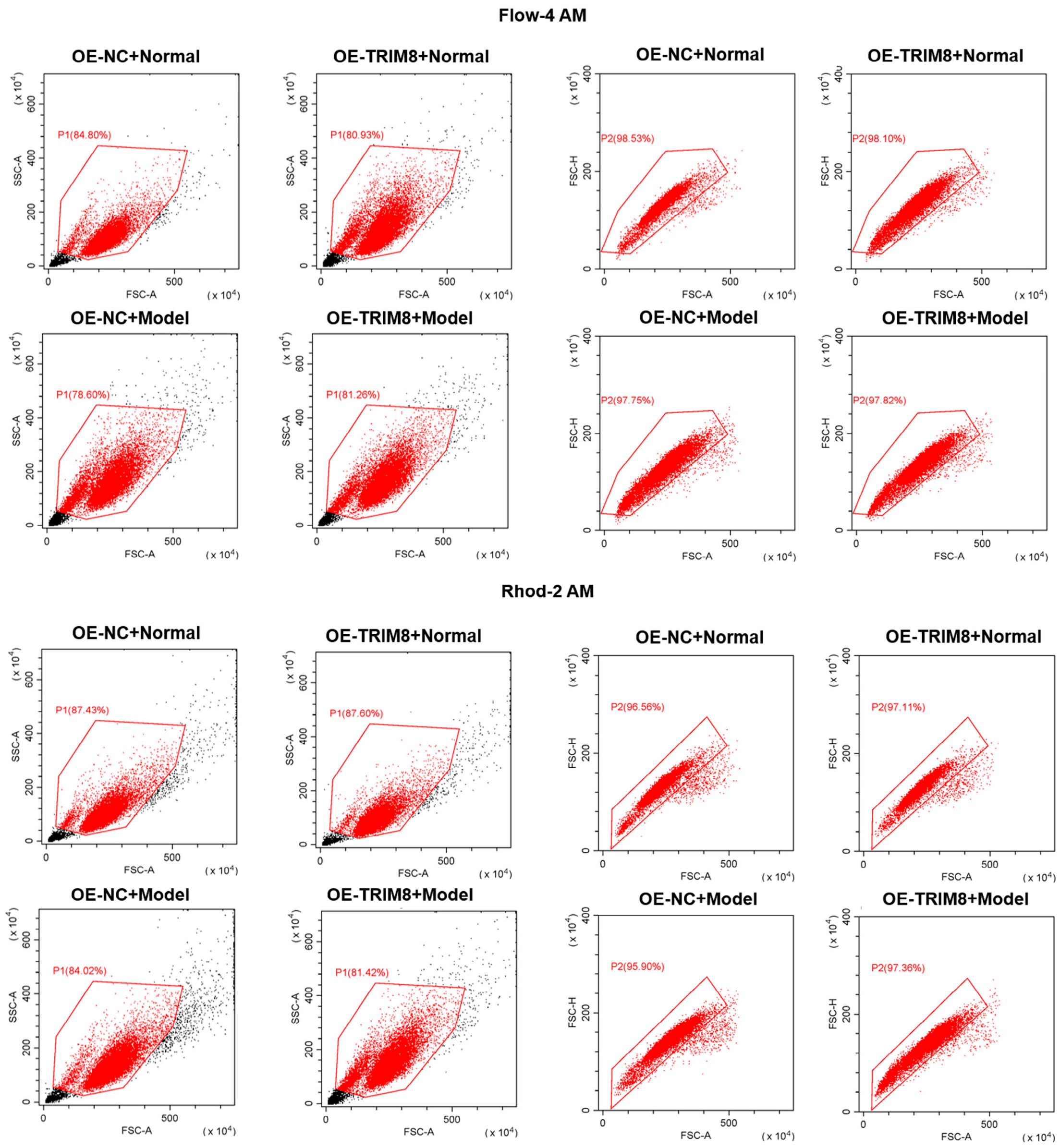

Supplementary Figure S1. Representative two-parameter FACS plots showing the gating strategy for intracellular calcium analysis in the TRIM8-overexpression experiment. Fluo-4 AM was used to detect cytosolic Ca^{2+}, and Rhod-2 AM was used to detect mitochondrial Ca^{2+}. Debris was excluded based on FSC-A versus SSC-A plots, and doublets or cell aggregates were excluded using FSC-A versus FSC-H plots. The mean fluorescence intensity (MFI) was calculated from the final gated single-cell population.

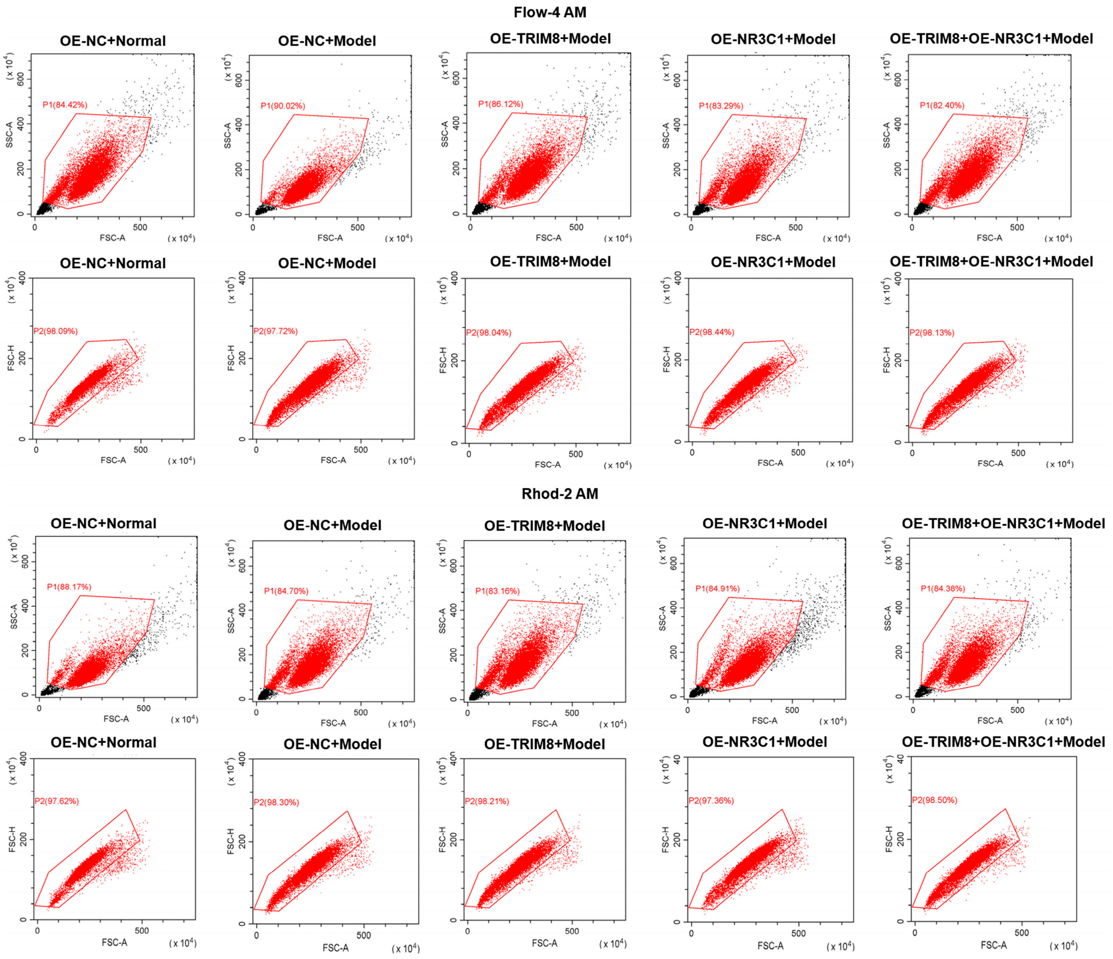

Supplementary Figure S2. Representative two-parameter FACS plots showing the gating strategy for intracellular calcium analysis in the NR3C1 rescue experiment. Fluo-4 AM was used to detect cytosolic Ca^{2+}, and Rhod-2 AM was used to detect mitochondrial Ca^{2+}. Debris was excluded based on FSC-A versus SSC-A plots, and doublets or cell aggregates were excluded using FSC-A versus FSC-H plots. The mean fluorescence intensity (MFI) was calculated from the final gated single-cell population.

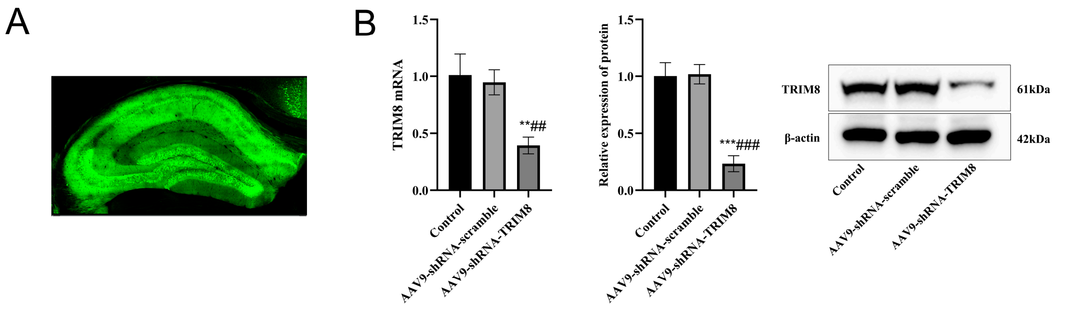

Supplementary Figure S3. Expression and localization of TRIM8 protein in hippocampal tissues of epileptic mice. (A) The expression of eGFP in brain tissue; (B) Expression of TRIM8 mRNA and protein in the right hippocampal tissues ($n = 3$). Compared with the Control group, * $p < 0.05$, ** $p < 0.01$, *** $p < 0.001$; compared with the AAV9-shRNA-scramble group, $^{\#} p < 0.05$, $^{\#\#} p < 0.01$, $^{\#\#\#} p < 0.001$.

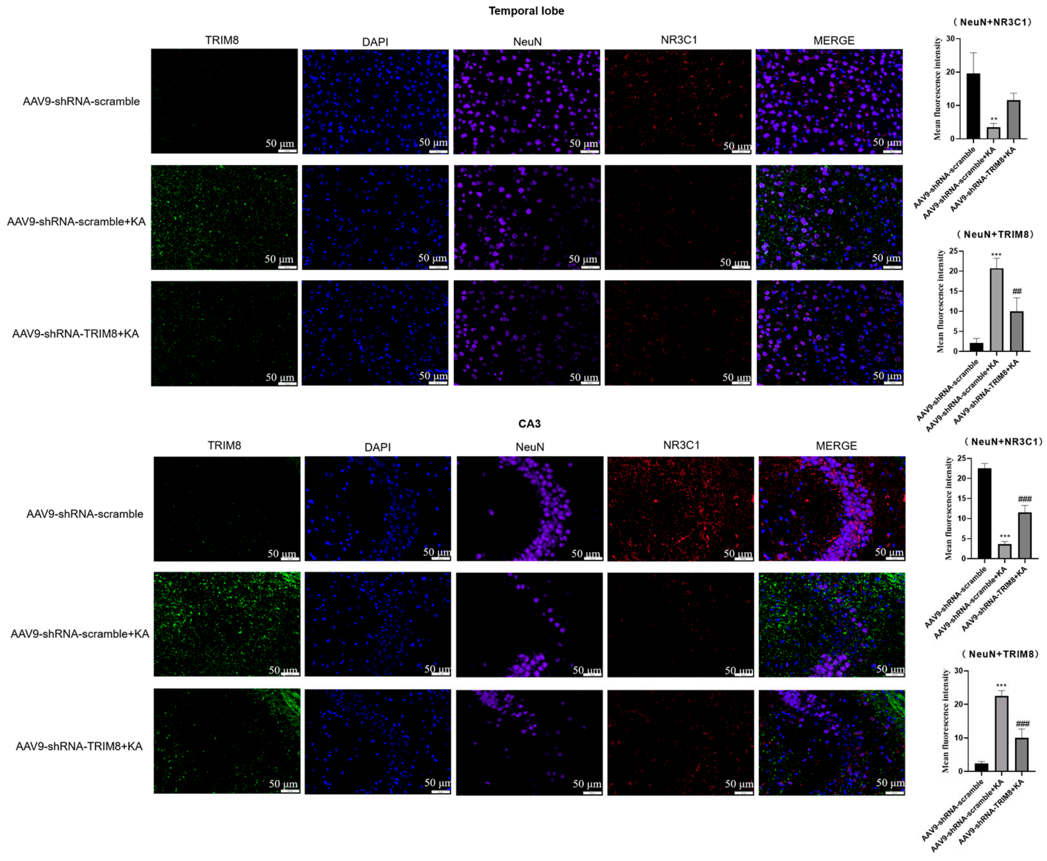

Supplementary Figure S4. Triple immunofluorescence staining for NeuN, TRIM8, and NR3C1 was performed to evaluate the expression of TRIM8 and NR3C1 in NeuN+ neurons in the CA3 region of the right hippocampus and the temporal lobe (n = 3). Compared with the AAV9-shRNA-scramble group, * $p < 0.05$, ** $p < 0.01$, *** $p < 0.001$; compared with the AAV9-shRNA-scramble + KA group, # $p < 0.05$, ## $p < 0.01$, ### $p < 0.001$.

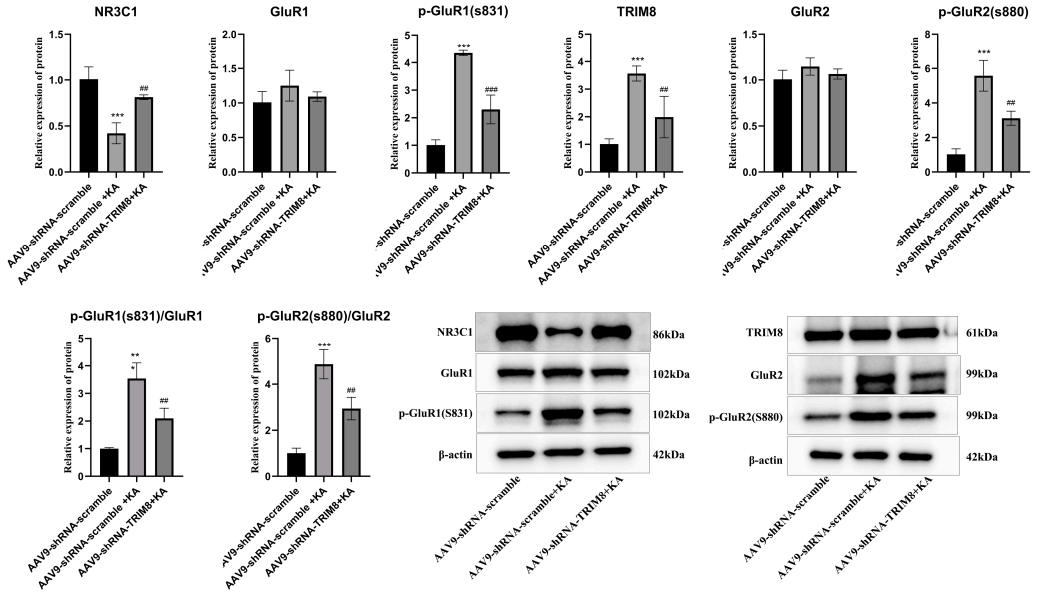

Supplementary Figure S5. The expression of TRIM8, NR3C1, GluR1, p-GluR1 (S831), GluR2, and p-GluR2 (S880) in the right hippocampus tissue (n = 3). Compared with the AAV9-shRNA-scramble group, * $p < 0.05$, ** $p < 0.01$, *** $p < 0.001$; compared with the AAV9-shRNA-scramble + KA group, # $p < 0.05$, ## $p < 0.01$, ### $p < 0.001$.
